# Human Nav1.6 Channels Generate Larger Resurgent Currents than Human Nav1.1 Channels, but the Navβ4 Peptide Does Not Protect Either Isoform from Use-Dependent Reduction

**DOI:** 10.1371/journal.pone.0133485

**Published:** 2015-07-16

**Authors:** Reesha R. Patel, Cindy Barbosa, Yucheng Xiao, Theodore R. Cummins

**Affiliations:** 1 Program in Medical Neuroscience, Indiana University School of Medicine, Indianapolis, Indiana, United States of America; 2 Paul and Carole Stark Neurosciences Research Institute, Indiana University School of Medicine, Indianapolis, Indiana, United States of America; 3 Department of Pharmacology and Toxicology, Indiana University School of Medicine, Indianapolis, Indiana, United States of America; University of Waterloo, CANADA

## Abstract

Voltage-gated sodium channels are responsible for the initiation and propagation of action potentials (APs). Two brain isoforms, Nav1.1 and Nav1.6, have very distinct cellular and subcellular expression. Specifically, Nav1.1 is predominantly expressed in the soma and proximal axon initial segment of fast-spiking GABAergic neurons, while Nav1.6 is found at the distal axon initial segment and nodes of Ranvier of both fast-spiking GABAergic and excitatory neurons. Interestingly, an auxiliary voltage-gated sodium channel subunit, Navβ4, is also enriched in the axon initial segment of fast-spiking GABAergic neurons. The C-terminal tail of Navβ4 is thought to mediate resurgent sodium current, an atypical current that occurs immediately following the action potential and is predicted to enhance excitability. To better understand the contribution of Nav1.1, Nav1.6 and Navβ4 to high frequency firing, we compared the properties of these two channel isoforms in the presence and absence of a peptide corresponding to part of the C-terminal tail of Navβ4. We used whole-cell patch clamp recordings to examine the biophysical properties of these two channel isoforms in HEK293T cells and found several differences between human Nav1.1 and Nav1.6 currents. Nav1.1 channels exhibited slower closed-state inactivation but faster open-state inactivation than Nav1.6 channels. We also observed a greater propensity of Nav1.6 to generate resurgent currents, most likely due to its slower kinetics of open-state inactivation, compared to Nav1.1. These two isoforms also showed differential responses to slow and fast AP waveforms, which were altered by the Navβ4 peptide. Although the Navβ4 peptide substantially increased the rate of recovery from apparent inactivation, Navβ4 peptide did not protect either channel isoform from undergoing use-dependent reduction with 10 Hz step-pulse stimulation or trains of slow or fast AP waveforms. Overall, these two channels have distinct biophysical properties that may differentially contribute to regulating neuronal excitability.

## Introduction

Voltage-gated sodium channels (VGSCs) mediate the inward current underlying the rising phase of the action potential and are consequently key regulators of excitability. These channels are comprised of a principal α subunit encoded by nine genes that associate covalently and non-covalently with one or more auxiliary β subunits encoded by four genes [1]. Three isoforms of VGSCs are highly expressed in the adult rodent central nervous system including: Nav1.1, Nav1.2 and Nav1.6 [2]. In this study, we focused on Nav1.1 and Nav1.6 because of their distinct cellular and subcellular localization. Specifically, Nav1.1 is predominantly found in parvalbumin-positive GABAergic neurons at detectable levels in the soma and proximal axon initial segment [3–5]. In contrast, Nav1.6 is found in both GABAergic and excitatory neurons within the soma, dendrites, nodes of Ranvier and distal axon initial segment [4, 6]. The axon initial segment is a key feature of neurons containing a high density of VGSCs and is the site of AP initiation [7–9]. These two channel isoforms are thought to have minimal overlap within the axon initial segment suggesting that they have distinct functions [4, 10]. It has previously been shown that different VGSC isoforms can play specific roles within the axon initial segment [11].

Hu *et al*. found that the high-threshold Nav1.2, expressed in the proximal axon initial segment of excitatory neurons, regulates the backpropagation of APs into somato-dendritic compartments while the low-threshold Nav1.6 determines the threshold for firing an AP that will propagate down the axon. In parvalbumin-positive GABAergic neurons, Ogiwara *et al*. found that Nav1.1 is important for the maintenance but not initiation of fast-firing. However, the full extent to which Nav1.1 and Nav1.6 contribute to sustaining high frequency firing is unclear.

Interestingly, one of the four auxiliary β subunits of VGSCs, Navβ4, is also enriched at the axon initial segment and nodes of Ranvier in many neuronal populations that have high frequency firing characteristics [12]. The C-terminal tail of Navβ4 has been proposed to act as an open-channel blocker that blocks the channel in the open state and upon repolarization unbinds to elicit a resurgence of sodium current, termed resurgent sodium current, after which the channels inactivate or deactivate [13, 14]. Resurgent sodium current is thus an atypical sodium current that occurs near threshold potentials immediately following an action potential spike. These currents were first identified in cerebellar Purkinje neurons, a type of parvalbumin-positive GABAergic neuron, and since have been observed in many neuronal populations [15–19]. Castelli *et al*. found that resurgent sodium current generation by pyramidal neurons in the perirhinal cortex could be abolished by focal application of TTX to the proximal axon, likely the axon initial segment. It is predicted that resurgent sodium current generation would enhance cellular excitability by providing a depolarizing drive after an AP spike to approach threshold for firing another AP [20–22].

To date, there has been no extensive comparison of the biophysical properties of Nav1.1 and Nav1.6, which is critical to understanding how these two channel isoforms could potentially contribute to the high frequency firing characteristics of parvalbumin-positive GABAergic neurons. The aims of this study were to 1) directly compare the biophysical properties of human Nav1.1 and Nav1.6, 2) determine whether resurgent sodium currents alter sodium influx in response to different AP waveforms that are characteristic of different cell types and 3) determine if Navβ4 peptide protects channels from undergoing use-dependent reduction. We found that these channel isoforms have distinct biophysical properties that could contribute to different characteristics of VGSCs important for fast-firing. Moreover, resurgent sodium current generation increases sodium influx in response to different duration AP waveforms, and while Navβ4 peptide enhances apparent recovery from inactivation, it does not protect channels from undergoing use-dependent reduction. These findings provide novel insight into the potential roles of these two channel isoforms as well as the potential role of Navβ4 peptide in maintaining a fast-firing phenotype.

## Materials and Methods

### cDNA Constructs

Optimized human constructs for Nav1.1 and Nav1.6 were designed in-house, purchased from Genscript (Piscataway, NJ) and subsequently hNav1.1 was subcloned into pTarget using XhoI and SalI restriction sites and hNav1.6 was subcloned into pcDNA3.1+ using KpnI and XbaI restriction sites. The amino acid sequences for the synthesized, human Nav1.1 and Nav1.6 cDNA constructs correspond with BAC21102.1 and NP_055006.1 in the NCBI database, respectively.

### Cell Cultures and Transfections

The use of HEK293T cells [23] was approved by the Institutional Biosafety Committee and followed the ethical guidelines for the National Institutes of Health for the use of human-derived cell lines. HEK293T cells were grown under standard tissue culture conditions (5% CO2; 37°C) with DMEM supplemented with 10% fetal bovine serum. HEK293T cells were transiently transfected using the calcium phosphate precipitation method. Briefly, a calcium phosphate-DNA mixture (4.5 μg channel construct and 0.5 μg EGFP) was added to cells in serum-free media for 4–5 hours and subsequently washed with fresh media. 12–24 hours post-transfection, cells were split onto laminin-coated glass coverslips. Cells were identified by expression of EGFP using a fluorescent microscope and whole-cell patch clamp recordings were obtained 36–72 hours post-transfection.

### Whole-Cell Patch Clamp Recordings

Whole-cell patch clamp recordings were obtained at room temperature (~23°C) using a HEKA EPC-10 amplifier, and the Pulse program (v 8.80, HEKA Electronic, Germany) was used for data acquisition. Voltage-clamp data (except for that obtained with AP waveforms) were digitized at 20 kHz and filtered at 5 kHz. Electrodes were fabricated from 1.7 mm capillary glass and fire-polished to a resistance of 0.9–1.3 MΩ using a Sutter P-97 puller (Sutter Instrument Company, Novato, CA). All voltage protocols were started 5 minutes after obtaining a gigaΩ seal and entering the whole-cell configuration, which controlled for time-dependent shifts in channel properties and allowed time for diffusion of Navβ4 peptide when used. Voltage errors were minimized to less than 5 mV using series resistance compensation and passive leak currents were cancelled by P/-5 subtraction. The bath solution contained in (mM): 140 NaCl, 1 MgCl2, 3 KCl, 1 CaCl2, and 10 Hepes, adjusted to a pH of 7.30 with NaOH. The pipette solution contained in (mM): 140 CsF, 10 NaCl, 1.1 EGTA, and 10 Hepes, adjusted to a pH of 7.30 with CsOH. Fluoride was used in part because it increased the stability of the recordings over time. Fluoride can also reduce persistent sodium current components [24], which enhanced our ability to compare resurgent current amplitudes. To induce resurgent currents in HEK293T cells, 200μM Navβ4 peptide (KKLITFILKKTREK-OH) (Biopeptide Co., San Diego, CA), a peptide that corresponds to part of the sequence of the C-terminal tail of the full-length Navβ4 subunit, was included in the pipette solution when specified. Resurgent currents are not detectable in HEK293T cells without Navβ4 peptide in the pipette solution.

### Modeling AP Waveforms

Fast spiking interneurons have substantially narrower AP waveforms than pyramidal neurons [25, 26], therefore we developed fast and slow AP waveforms to elicit currents in voltage-clamp experiments. The AP waveforms were initially generated in the NEURON simulation environment [27] using previously developed computational models of pyramidal and fast-spiking GABAergic neurons [28]. The slow (pyramidal) and fast (fast-spiking GABAergic) waveforms were then digitally modified to have identical initial membrane potentials, peak potentials and after-hyperpolarization potentials. Thus, the major differences were the rate of rise, the rate of repolarization and the overall duration of the AP. The mid-height duration of the fast AP waveform (modeled at 37°C) was 0.29 ms and that of the slow AP waveform was 1.0 ms, which are similar to the durations measured for fast spiking cortical interneurons and cortical pyramidal neurons (Table 1) [25]. Since voltage-clamp experiments were carried out at room temperature (~23°C), the waveforms were adjusted to account for channel gating at room temperature. Mammalian sodium channel kinetics as well as AP duration have temperature coefficients (Q10s) of approximately 2 [29–31]. Therefore, AP waveforms were slowed by a factor of 2.5 (Q10 = 2). Parameters for modeled (37°C) and actual (23°C) action potential waveforms are summarized in Table 1. Trains of action potentials were created by concatenation of 20 single action potential waveforms. The initial trains had a spike frequency of 100 Hz. These were modified to also generate 25 Hz trains by lengthening the interspike interval using linear voltage ramps. To account for recording at room temperature, the 25 and 100 Hz trains were scaled using a Q10 of 2. The effective frequencies after scaling were 8 and 33 Hz (S1 Fig). The scaled AP waveforms and trains of APs were then used as voltage-command waveforms to elicit sodium currents. Data collected from AP voltage command waveforms represent an average of five traces. Data obtained with 33 Hz AP waveform trains were acquired at 6.6 kHz and filtered at 3.3 kHz and those obtained with 8 Hz waveform trains were acquired at 4 kHz and filtered at 2 kHz.

**Table 1:** Modeled Action Potential Parameters.

	Slow AP		Fast AP	
	Modeled 37°C	Actual 23°C	Modeled 37°C	Actual 23°C
**Max rate of rise (V/s)**	542.5	180.8	1234.0	411.3
**Max rate of decay (V/s)**	-109.5	-36.5	-367.5	-122.5
**Overshoot Amplitude (mV)**	39.3	39.3	39.3	39.3
**Width at -20mV (ms)**	1.0	3.0	0.29	0.87
**Peak AHP (mV)**	-84.9	-84.9	-84.9	-84.9

### Data Analysis

Electrophysiological data were analyzed using Pulsefit (v 8.67 HEKA Electronic, Germany), Microsoft Excel, Origin (v 8.0, OriginLab Corp, Northhampton, MA), and Prism (v 6.0, Graphpad Software Inc., La Jolla, CA). Steady-state activation and inactivation curves were fit to a Boltzmann function to obtain midpoint and slope values. Quantification of the area under the curve was measured from 20ms to 35ms and baseline was set to zero. All data points are presented as mean ± SEM and *n* is the number of experimental cells from which recordings were obtained. Statistical significance was assessed using an unpaired t-test or a one-way ANOVA when indicated.

## Results

### hNav1.1 and hNav1.6 have differential biophysical properties

**Fig 1:**
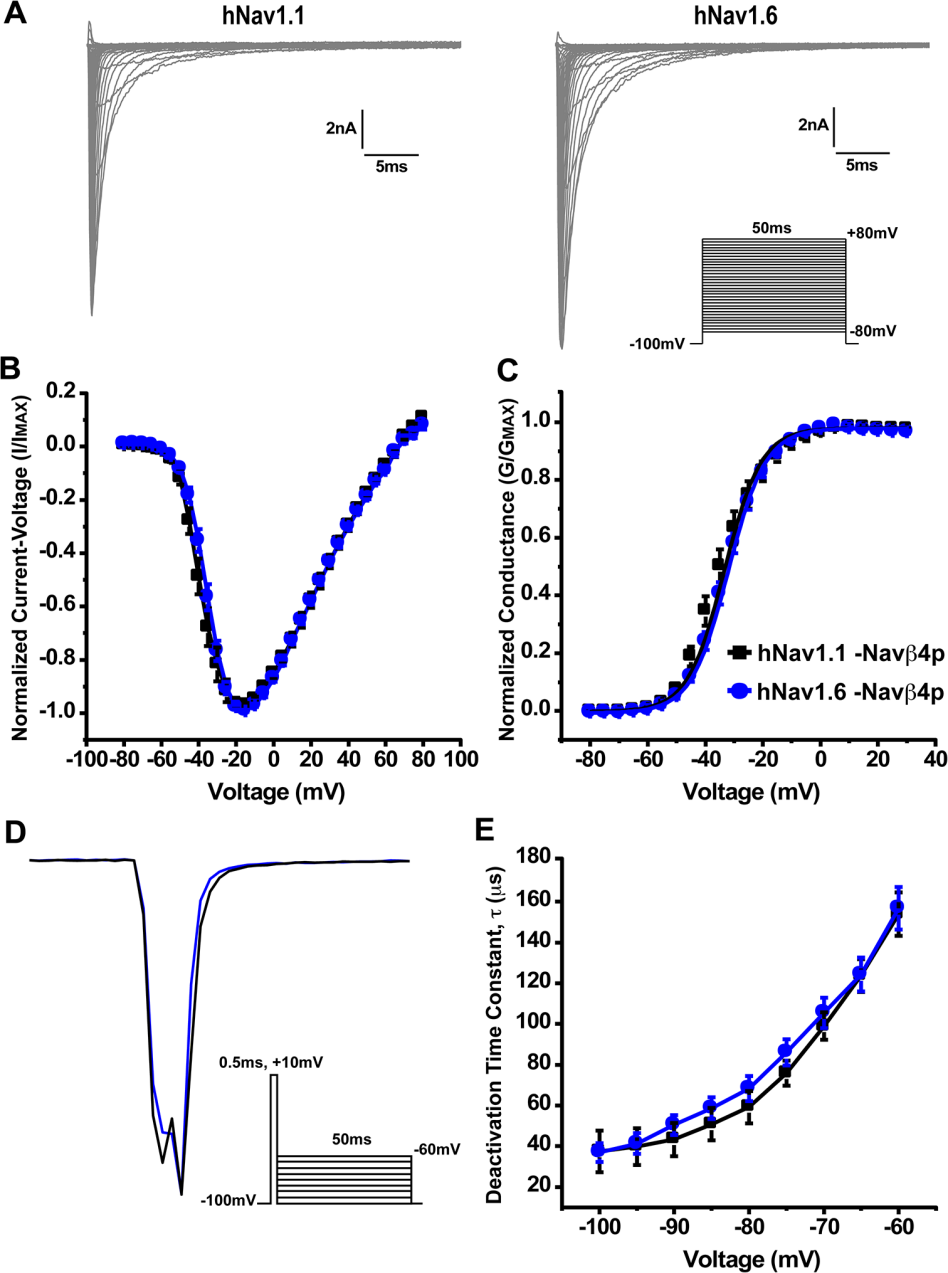
Current-voltage relationship, voltage-dependence of steady-state activation and deactivation kinetics of hNav1.1 and hNav1.6. A, Representative current traces recorded from hNav1.1 (left) and hNav1.6 (right) expressed in HEK293T cells. The currents were elicited by applying 50ms step-depolarization to potentials ranging from -80mV to +80mV from a holding potential of -100mV. *Inset*, Protocol used to obtain current-voltage traces. B, Normalized peak current-voltage relationship for hNav1.1 (black squares; n = 14) and hNav1.6 (blue circles; n = 14). C, Voltage-dependence of steady-state activation shows no difference in conductance between hNav1.1 and hNav1.6. D, Representative traces showing hNav1.1 (black) and hNav1.6 (blue) deactivation tail currents at -70mV. *Inset*, Protocol used to elicit deactivation tail currents. E, Time constants of channel deactivation were similar for hNav1.1 and hNav1.6 at voltages ranging from -100mV to -60mV. Time constants were obtained by a brief 0.5ms depolarization to +10mV followed by a series of repolarizations to potentials from -100mV to -60mV eliciting tail currents that were fit to a single exponential function.

To compare the biophysical properties of human Nav1.1 and Nav1.6 (hNav1.1 and hNav1.6), we transiently transfected HEK293T cells with each channel isoform and obtained whole-cell patch clamp recordings. hNav1.1 and hNav1.6 have a similar current-voltage relationship for peak sodium current (Fig 1A and 1B). Correspondingly, these two channel isoforms show no differences in their voltage-dependence of steady-state activation (Fig 1C). Gating parameters are summarized in Table 2. We then examined the kinetics of deactivation by applying a brief (0.5ms) depolarizing step pulse to +10mV followed by a repolarizing step to voltages ranging from -60mV to -100mV for 50ms eliciting tail currents that were fit to a single exponential function (Fig 1D, inset). Representative traces of deactivation tail current can be seen in Fig 1D. The time constants for the kinetics of deactivation were similar for both channel isoforms across all voltages tested (Fig 1E).

**Table 2:** Summary of activation and inactivation gating parameters.

	Activation		Inactivation		
	V1/2	Slope	V1/2	Slope	n
**hNav1.1**	-34.8 ± 1.8	6.7 ± 0.3	-67.2 ± 1.7	4.7 ± 0.1	14
**hNav1.6**	-32.6 ± 1.1	6.5 ± 0.1	-71.9 ± 1.3*	5.9 ± 0.1*	14

* p < 0.05 Compared to hNav1.1

**Fig 2:**
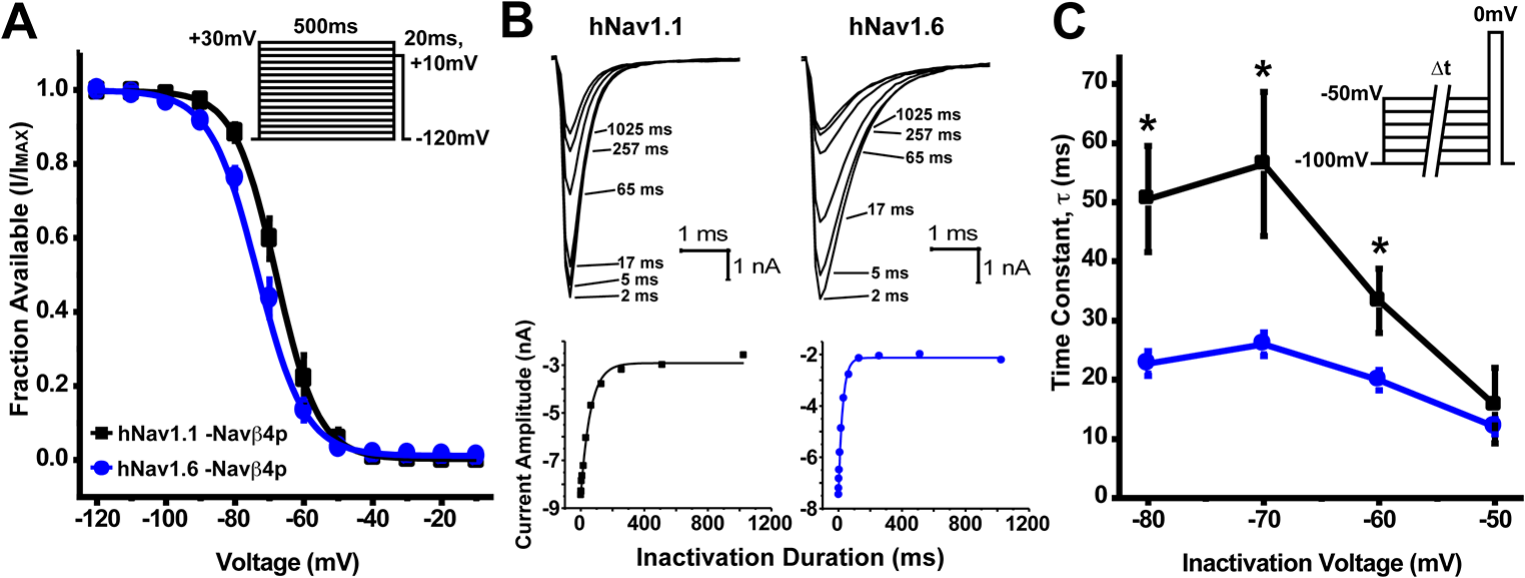
Voltage-dependence of steady-state inactivation and kinetics of development of closed-state inactivation for hNav1.1 and hNav1.6. A, To examine the voltage-dependence of steady-state fast inactivation a series of 500ms steps from -120mV to +30mV followed by a 20ms step pulse to +10mV was used to measure channel availability. Midpoints of the voltage-dependence of steady-state fast inactivation were estimated by fitting data with a Boltzmann function and was more hyperpolarized for hNav1.6 (blue circles; *n* = 14) compared to hNav1.1 (black squares; *n* = 14)(Unpaired t-test, p < 0.05). Table 2 summarizes gating parameters. *Inset*, Protocol used to measure steady-state inactivation. B, Top: Representative family of current traces generated by hNav1.1 (left) and hNav1.6 (right) showing the rate of development of inactivation at -70mV. Bottom: Plots for the time course of development of inactivation for the peak current from the corresponding cell above. C, Time constants for development of closed-state inactivation are smaller for hNav1.6 (blue circles; *n* = 8) compared to hNav1.1 (black squares; *n* = 9) (Unpaired t-test, *p < 0.05). Time constants were determined by single exponential fits to time courses measured using the voltage protocol depicted in the inset.

Next, we examined the voltage-dependence of steady-state inactivation by holding cells at voltages ranging from -120mV to +30mV for 500ms and then applying a 20ms test pulse to +10mV to determine the fraction available (Fig 2A, inset). hNav1.6 had a small but significant hyperpolarizing shift in the midpoint (-72 ± 1mV; *n* = 14) of the voltage-dependence of steady-state inactivation curve compared to hNav1.1 (-67 ± 2mV; *n* = 14) (Fig 2A, Table 2). To explore what was underlying the shift in inactivation we examined the development of closed-state inactivation, which reflects the direct transition of channels from a closed into an inactivated state. Development of closed-state inactivation was measured using a voltage protocol in which cells were held at -100mV and stepped to a prepulse potential ranging from -100mV to -50mV for increasing durations immediately followed by a test pulse to 0mV to assess the fraction of current inactivated during the prepulse (Fig 2C, inset). The data were plotted (peak current amplitude as a function of inactivation duration) and fit to a single exponential function to determine the time constants for development of closed state inactivation as seen in Fig 2B. We found that hNav1.6 had statistically smaller tau values for development of closed state inactivation at voltages ranging from -80mV to -60mV compared to hNav1.1 (Fig 2C). Specifically, at voltages near typical resting potentials in neurons such as -70mV, hNav1.1 (τ = 56.5 ± 12.1ms; *n* = 9) had a slower rate of closed-state inactivation development compared to hNav1.6 (τ = 26.0 ± 2.0ms; *n* = 8), which would allow it to be more resistant to inactivation during slow sub-threshold depolarizations [32]. This would result in greater channel availability for hNav1.1 to open once the membrane is depolarized to threshold potentials for firing an AP.

**Fig 3:**
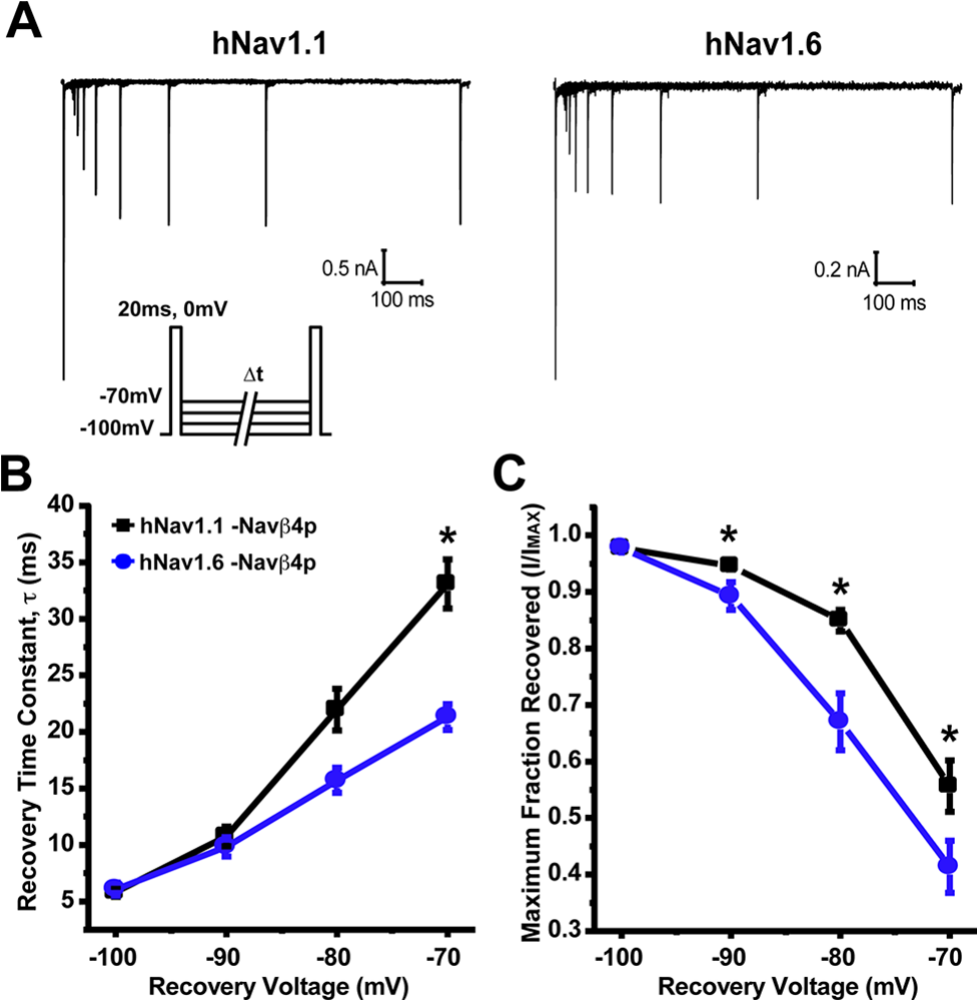
Rate and fraction of recovery from fast inactivation by hNav1.1 and hNav1.6. A, Representative traces of recovery from fast inactivation measured by first inducing fast inactivation from a holding potential of -100mV with a 20ms step pulse to 0mV and then applying a 20ms test pulse to 0mV subsequent to various recovery times at -70mV. *Inset*, Protocol used to measure recovery from fast inactivation. B, hNav1.6 (blue circles; *n* = 17) has a smaller time constant for recovery at -70mV compared to hNav1.1 (black squares; *n* = 19). C, Maximal fraction recovered from fast inactivation was greater for hNav1.1 at voltage ranging from -90mV to -70mV compared to hNav1.6 (Unpaired t-test, *p < 0.05).

We also compared recovery from fast inactivation (repriming), a property that can limit the channels ability to sustain high firing frequencies. To do this, we held cells at -100mV and applied a prepulse to 0mV for 20ms to induce fast inactivation and then allowed the channels to recover for increasing durations at potentials ranging from -100mV to -70mV before applying a test pulse to 0mV for 20ms to measure the available channels (Fig 3A, inset). Representative traces of recovery from fast inactivation at -70mV are depicted in Fig 3A. Each test pulse current was normalized to the maximal current at each time point and plotted as a function of time for each recovery voltage (S2 Fig). Time constants for repriming kinetics were estimated using single exponential fits and these time constants are plotted as a function of recovery voltage in Fig 3B. We found that hNav1.1 and hNav1.6 had similar time constant values for recovery from inactivation at voltages ranging from -100mV to -80mV. However, at -70mV hNav1.6 (τ = 22.3 ± 1.1ms; *n* = 17) had significantly faster repriming kinetics compared to hNav1.1 (τ = 33.1 ± 2.1ms; *n* = 19). It is important to note that the maximal fraction recovered was greater for hNav1.1 compared to hNav1.6 at recovery voltages from -90mV to -70mV (Fig 3C), consistent with the differences observed in the voltage-dependence of steady-state inactivation.

### hNav1.6 has a greater propensity to generate resurgent currents than hNav1.1

**Fig 4:**
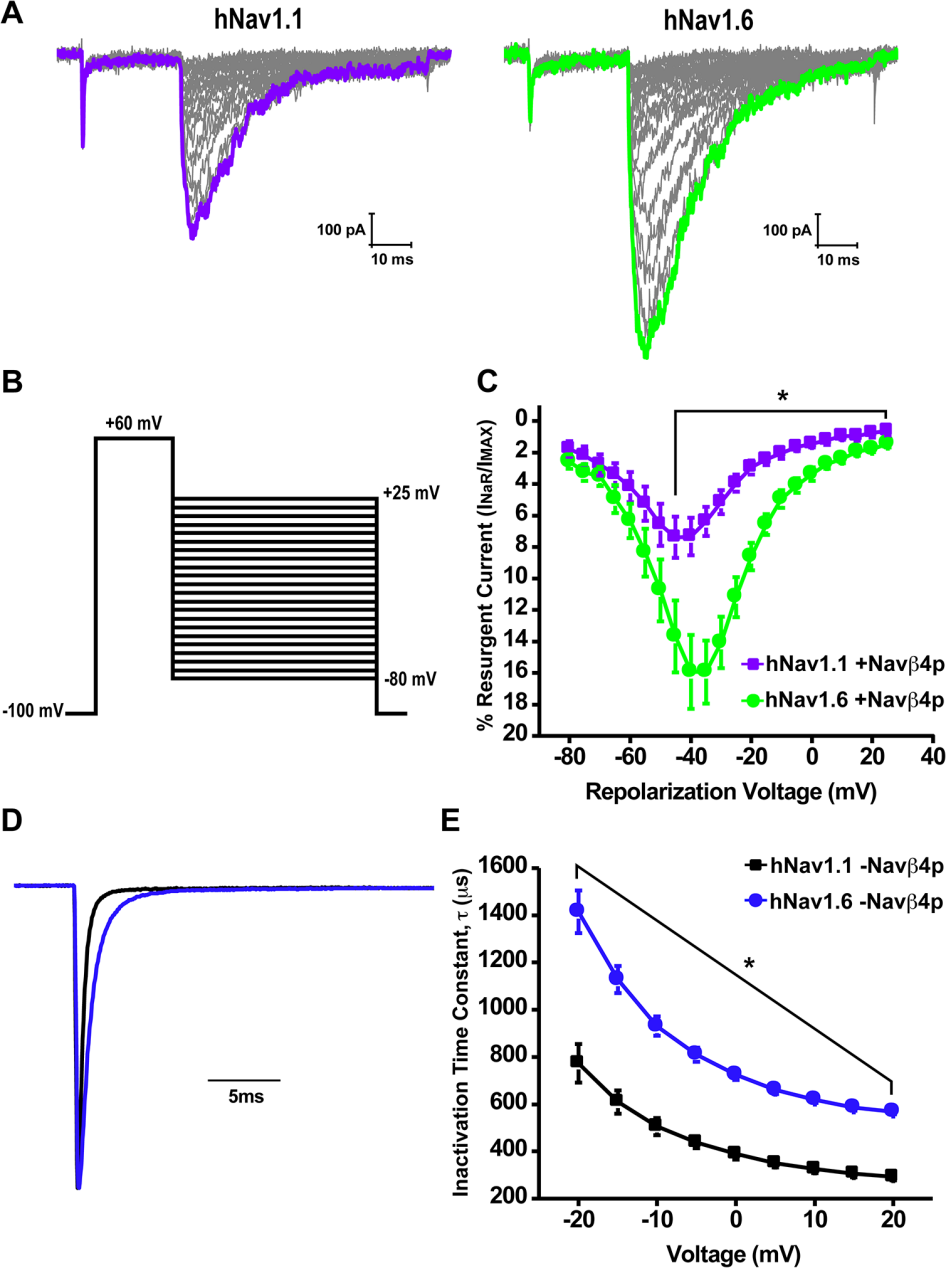
Resurgent current generation and kinetics of inactivation of hNav1.1 and hNav1.6. A, Representative family of resurgent current traces from hNav1.1 (left) and hNav1.6 (right) with Navβ4 peptide in the pipette solution. Currents were elicited by depolarization to +60mV for 20ms followed by repolarization to potentials ranging from +25mV to -80mV for 50ms. B, Protocol used to measure resurgent currents. C, Current-voltage curve of peak resurgent current normalized to peak transient current measured at +10mV by hNav1.1 (purple squares; *n* = 15) and hNav1.6 (green circles; *n* = 15). D, Representative normalized current traces elicited by a step depolarization from -100mV to -10mV by hNav1.1 (black) and hNav1.6 (blue) without Navβ4 peptide in the pipette solution. E, Averaged decay time constants measured at potentials ranging from -20mV to +20mV from hNav1.1 (black square; *n* = 14) and hNav1.6 (blue circles; *n* = 14) (Unpaired t-test, *p < 0.05).

We next asked whether there was an intrinsic difference in the ability of hNav1.1 and hNav1.6 to generate resurgent sodium currents. We examined resurgent current generation by hNav1.1 and hNav1.6 in HEK293T cells by inclusion of a peptide (Navβ4 peptide) corresponding to a portion of the C-terminal tail of the Navβ4 auxiliary subunit in the pipette solution, which has been previously shown to induce resurgent currents in this cell type [33–35]. Fig 4A shows a family of representative resurgent current traces generated from cells expressing either hNav1.1 or hNav1.6 obtained by applying an initial depolarizing step to +60mV for 20ms from a holding potential of -100mV followed by a 50ms step to repolarizing voltages ranging from +25mV to -80mV (Fig 4B). Resurgent currents were quantified by dividing the peak resurgent current amplitude measured after 1.5ms into the repolarizing step (to prevent contamination by tail currents) by the peak transient current measured with a 20ms test pulse at +10mV and are shown as a percentage of the peak transient current amplitude. hNav1.6 demonstrated a greater propensity to generate resurgent currents within voltages ranging from -45mV to +25mV compared to hNav1.1 (Fig 4C). The peak resurgent current was more than 2-fold greater for hNav1.6 (15.9 ± 2.4%; *n* = 15) compared to hNav1.1 (7.4 ± 1.3%; *n* = 15) and occurred at -35mV and -45mV, respectively.

One of the major determinants of resurgent current generation is the rate of inactivation because the Navβ4 peptide is thought to directly compete with the ability of the intrinsic fast inactivation gate to bind the channel. Therefore, we examined the kinetics of fast inactivation of peak transient sodium currents by fitting the decay phase of macroscopic currents elicited with test potential steps to voltages ranging from -20mV to +20mV with a single exponential function. Fig 4D shows representative normalized traces elicited by a voltage step from -100mV to -10mV demonstrating the slower decay phase of hNav1.6 compared to hNav1.1. We found that hNav1.6 has significantly larger time constants for kinetics of inactivation compared to hNav1.1 at all the voltages tested (Fig 4E). The slower kinetics of inactivation for hNav1.6 corresponded to its greater ability to generate resurgent currents. We did not observe any differences in the kinetics of inactivation with and without of Navβ4 peptide for either channel isoform (S3 Fig).

### Sodium influx through hNav1.1 and hNav1.6 in response to slow and fast AP waveforms is altered in the presence of Navβ4 peptide

**Fig 5:**
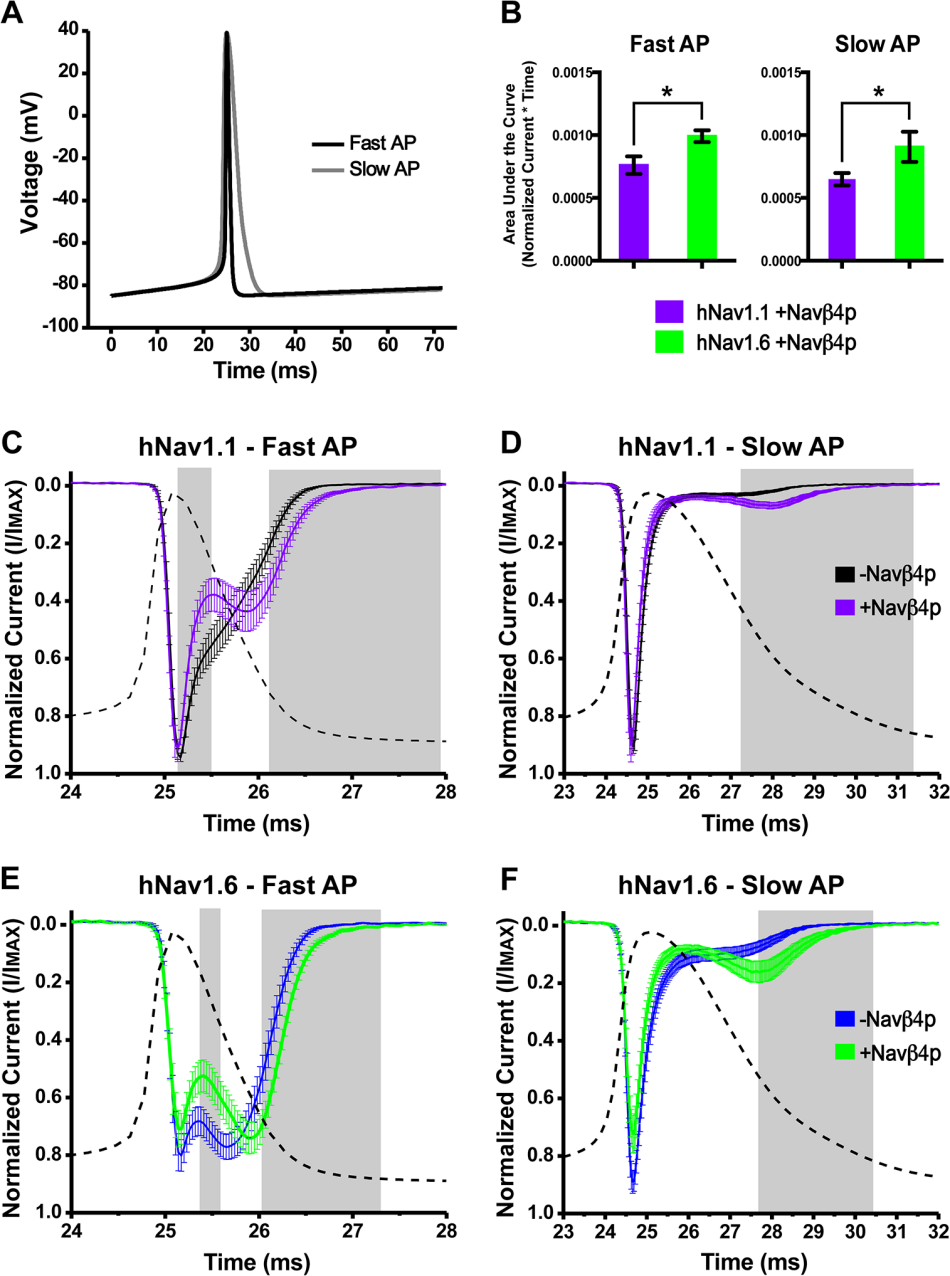
Sodium influx in response to fast and slow AP waveforms with and without Navβ4 peptide. A, Fast and slow voltage command waveforms modeled using NEURON. B, Area under the curve for currents elicited by the fast (left) and slow waveform (right) from cells expressing hNav1.1 (purple bars) and hNav1.6 (green bars) measured between 20 and 35ms (Unpaired t-test, *p < 0.05). Currents generated in response to fast and slow AP waveforms were normalized and then averaged. C, hNav1.1 generated sodium current in response to a fast voltage command waveform in the absence (black traces; *n* = 17) and presence (purple traces; *n* = 14) of Navβ4 peptide. D, Response of hNav1.1 to a slow voltage command waveform. E, hNav1.6 generated sodium current in response to a fast voltage command waveform in the absence (blue trace; *n* = 14) and presence (green trace; *n* = 15) of Navβ4 peptide. F, Response of hNav1.6 to a slow voltage command waveform. Grey boxes represent regions of statistically significant differences (Unpaired t-test, p < 0.05).

AP waveforms vary with cell type and one of the major differences is the width of the action potential [36, 37]. It is unknown if and where during the AP waveform resurgent sodium current generated by hNav1.1 or hNav1.6 would result in an increase in sodium influx. We therefore wanted to examine the influence of resurgent current generation on sodium influx in response to a slow and fast AP waveform. To do this, we first modeled AP waveforms from fast-spiking GABAergic (fast AP) and pyramidal (slow AP) neurons using the simulation program NEURON (Fig 5A) [27]. We adjusted the initial resting membrane potential, peak amplitude and the after-hyperpolarization potential of the AP waveforms to be identical in order to focus on implications of the difference in the AP width observed between these two different types of neurons. We then used the AP waveform as a voltage-command waveform to elicit sodium currents.

Fig 5C and 5E show the responses of hNav1.1 and hNav1.6 to the fast AP waveform (superimposed for comparison in a dotted line) as an average of normalized traces plotted versus time. Note that not all traces reach a value of one because the peak does not occur at the same exact time in each cell, therefore after averaging the normalized traces from each cell the averaged peak value is less than one. The second peak, or hump, in the decaying phase of the sodium current is likely generated by the fraction of channels that do not undergo fast inactivation during the upstroke of the action potential. hNav1.1 mediated sodium influx was significantly decreased between 25.2 and 25.5ms and increased during the second non-inactivating component of the current between 26.1 and 28ms by Navβ4 peptide, as highlighted by the grey shaded boxes. Similarly, hNav1.6 mediated sodium influx in the presence of Navβ4 peptide was significantly decreased between 25.4 and 25.6ms and increased between 26.0 and 27.3ms. The decrease in sodium influx likely reflects binding of Navβ4 peptide to the channel while the unbinding of Navβ4 peptide increased the duration of the second non-inactivating component. Responses to the slow AP waveform exhibited a smaller second non-inactivating component compared to the fast AP waveform. However, just as with the fast AP waveform, Navβ4 peptide significantly increased sodium influx during the second non-inactivating component for hNav1.1 between 27.3 and 31.4ms and hNav1.6 between 27.7 and 30.4ms, increasing both the duration of sodium influx and amplitude of the second non-inactivating component (Fig 5D and 5F). Unlike with the fast AP waveform, the sodium influx in response to the slow AP waveform did not show the same decrease in current following the initial peak in the presence of Navβ4 peptide. Overall, there was a statistically greater sodium influx mediated by hNav1.6 compared to hNav1.1 as measured by the area under the curve in the presence and absence of Navβ4 peptide with both the slow and fast AP waveforms (Fig 5B; S4 Fig).

### Navβ4 peptide mediated open-channel block allows channels to recover faster

**Fig 6:**
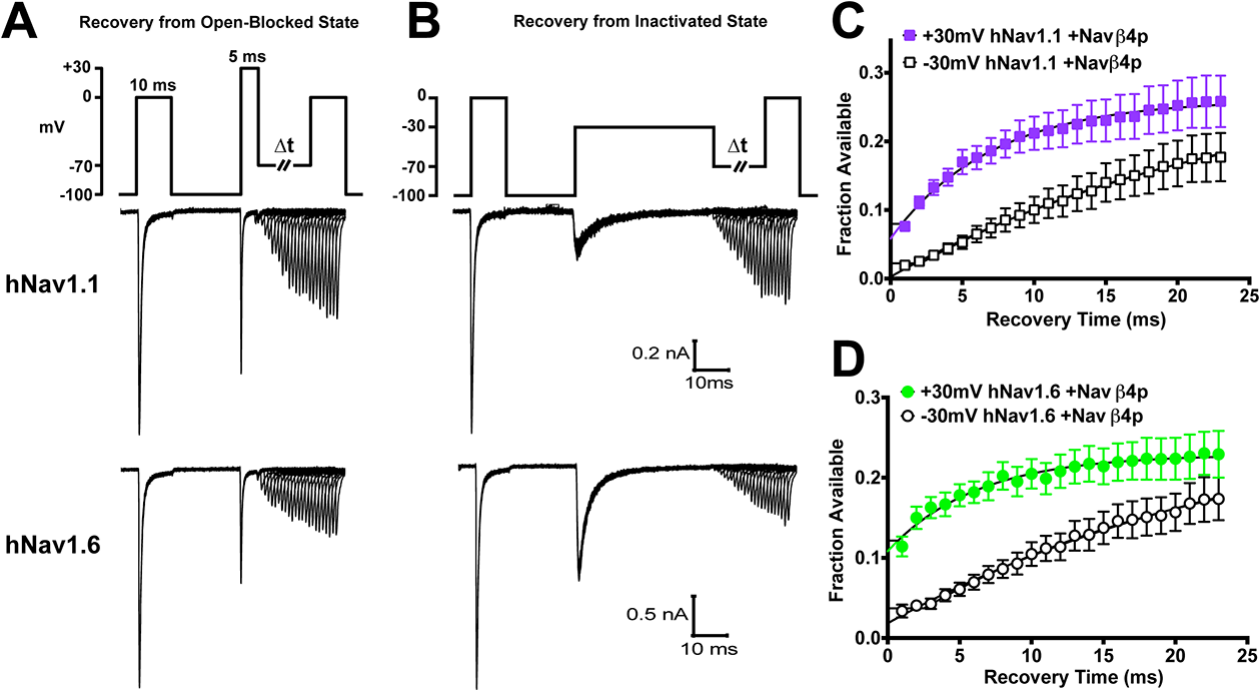
Recovery of channels from open-blocked versus inactivated states. A, Recovery at -70mV for increasing durations following a brief, 5ms pulse to +30mV to allow channels to enter an open-blocked state (top). Representative traces for the time courses of recovery generated by hNav1.1 (middle) and hNav1.6 (bottom). B, Recovery at -70mV for increasing durations following a 40ms pulse to -30mV to allow channels to enter an inactivated state. Representative traces for the time courses of recovery generated by hNav1.1 (middle) and hNav1.6 (bottom). C and D, Summary data showing the fraction available after a 5ms pulse to +30mV (filled) versus a 40ms pulse to -30mV (open) plotted against the recovery time for hNav1.1 (squares; *n* = 21) and hNav1.6 (circles; *n* = 14). Fraction available was calculated by normalizing the peak current elicited by the test pulse to the peak current elicited by a step depolarization from -100mV to 0mV at the beginning of the protocol. Data are fit to a single exponential function.

The open-channel blocker responsible for mediating the generation of resurgent current is thought to allow channels to bypass classic inactivation and enhance recovery [38]. We therefore tested whether Navβ4 peptide mediated open-channel block allows channels to recover faster. To do this we used two different voltage command protocols (adapted from Raman and Bean, 2001) in which we applied either a brief, 5ms pulse to +30mV or a longer, 40ms pulse to -30mV to accumulate channels in an open-blocked or inactivated state, respectively, and subsequently allowed channels to recover at -70mV for increasing durations before applying a test pulse to 0mV to measure the fraction of channels available (Fig 6A and 6B). We found that both hNav1.1 and hNav1.6 channels have smaller time constants following recovery from an open-blocked state (hNav1.1: τ = 7.0 ms; hNav1.6: τ = 6.2 ms) compared to an inactivated state (hNav1.1: τ = 26.1 ms; hNav1.6: τ = 28.5 ms) (Fig 6C and 6D). It is important to note that we did not observe any differences in the time course for recovery with these two protocols in the absence of Navβ4 peptide (S5 Fig), which indicates that in the absence of Navβ4 peptide the two distinct depolarizing pulses (5 ms at +30 mV versus 40 ms at -30 mV) induce similar inactivation states for the sodium channels.

### Navβ4 peptide does not protect hNav1.1 or hNav1.6 from use-dependent reduction with 10 Hz step-pulses

**Fig 7:**
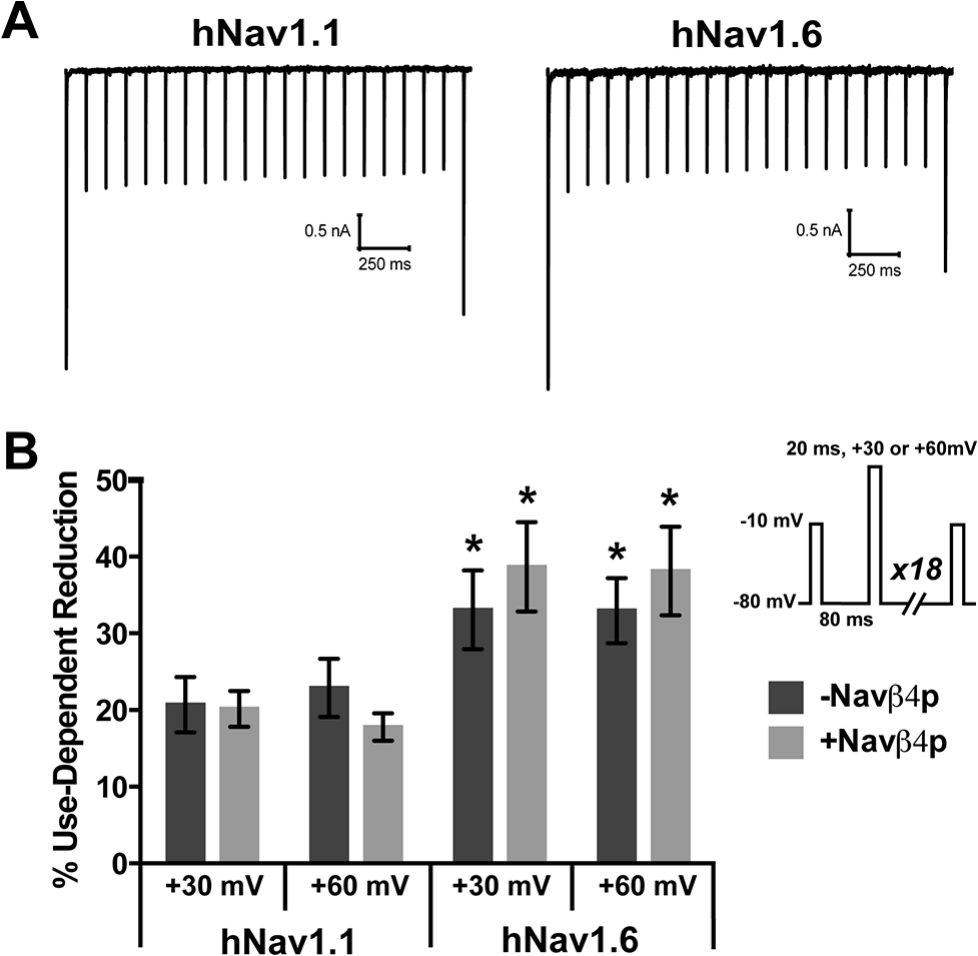
Effects of the Navβ4 peptide on use-dependent reduction with 10 Hz stimulation. A, Representative traces of use-dependent reduction traces generated by hNav1.1 (left) and hNav1.6 (right) when pulsed at +30mV. Use-dependent reduction was examined with an initial and final 20ms step pulse to -10mV from -80mV to assess the current available before and after 19 consecutive step depolarization from -80mV for 80ms to +30mV or +60mV for 20ms. B, Percent inhibition was calculated between the initial and final currents for cells in the absence (dark grey bars) and presence (light grey bars) of Navβ4 peptide (*n* = 8–9). *Inset*, Abbreviated protocol used to measure use-dependent reduction. *p < 0.05 compared to corresponding hNav1.1 group.

Since Navβ4 peptide mediated open-channel block enhances recovery, we predicted that the presence of the open channel blocker would allow channels to better follow a 10 Hz step-pulse stimulus by protecting channels from undergoing use-dependent reduction. We used a protocol in which we assessed the peak current with an initial 20ms step pulse to -10mV from -80mV followed by 19 step-depolarizations (at 10 Hz) from -80mV to either +30mV or +60mV for 20ms before a final test pulse to -10mV for 20ms to determine the remaining channels available (Fig 7A and 7B, inset). Two depolarization voltages were used because Navβ4 peptide binds more stably to the channel at higher depolarization voltages [39]. We calculated the percent current reduced between the initial and final test pulses and found that hNav1.6 undergoes approximately 15% more use-dependent reduction compared to hNav1.1 (Fig 7B). The presence of Navβ4 peptide did not alter use-dependent reduction with 10 Hz step-pulses to +30mV or +60mV by either channel isoform.

One possibility is that the use-dependent reduction in current was independent of fast-inactivation processes, but rather reflected slow inactivation of hNav1.1 and hNav1.6 channels. To evaluate entry into slow inactivated states during the 10 Hz trains, we measured the fraction of channels that recover from fast inactivation after the 10 Hz stimulus protocol by applying a 20ms step pulse to -120mV following the last step depolarization to +60mV (allowing channels to recover from fast but not slow inactivation) and used a subsequent test pulse to -10mV to measure channel availability. We found that both hNav1.1 (91.56 ± 1.6% availability) and hNav1.6 (112.3 ± 7.4% availability) nearly or completely recovered during this brief recovery pulse, suggesting channels (particularly hNav1.6 channels) are not undergoing slow inactivation.

**Fig 8:**
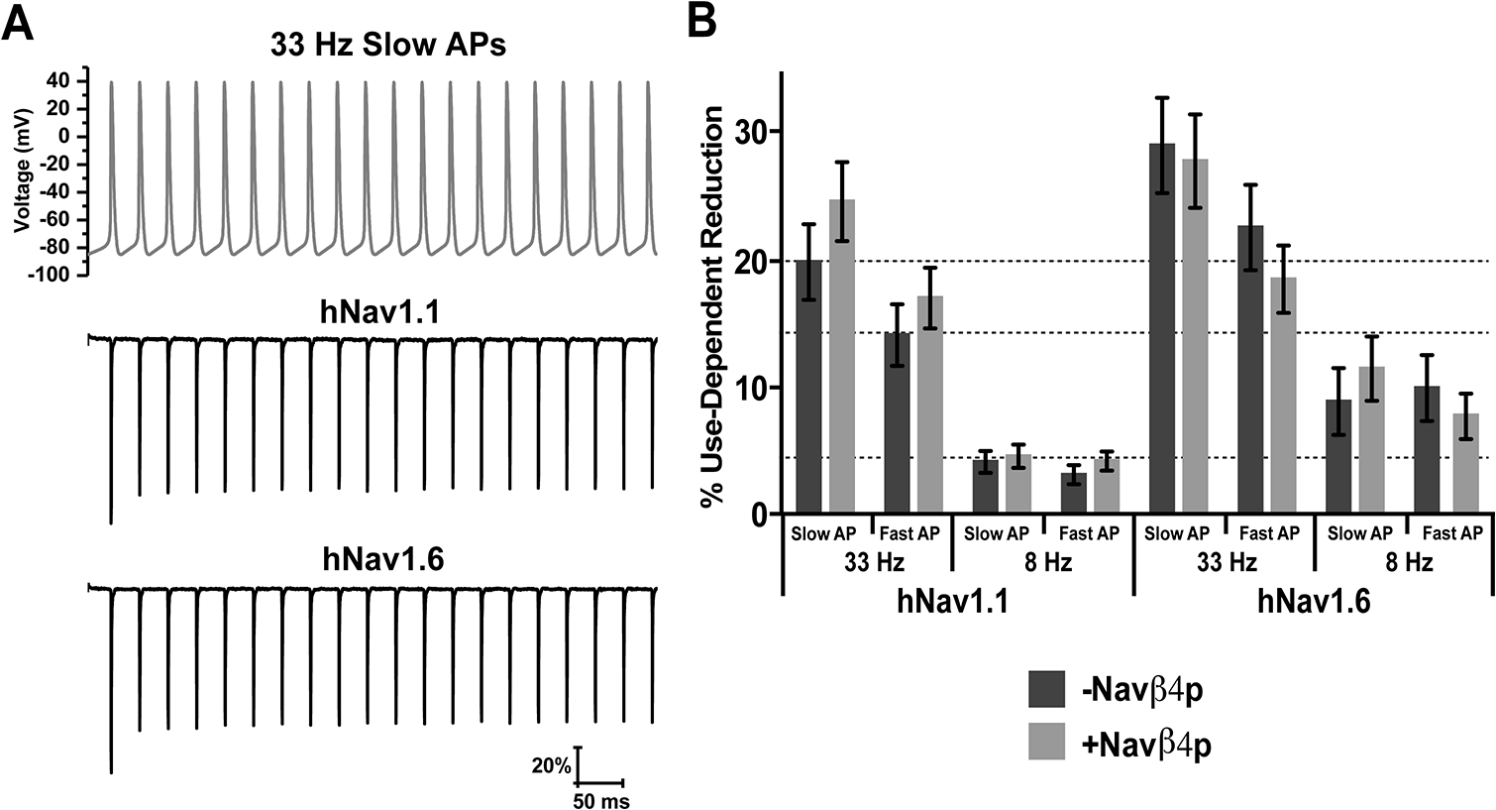
Effects of Navβ4 peptide on use-dependent inhibition with 8 and 33 Hz trains of slow or fast AP waveforms. A, Top: Voltage command waveform of 33 Hz slow APs comprising of a total of 20 APs. Below are representative traces of the response from cells expressing hNav1.1 (middle) and hNav1.6 (bottom) to the voltage command waveform. B, Percent reduction between the first and last current elicited by each frequency and waveform type in the absence (dark grey bars) and presence (light grey bars) Navβ4 peptide (*n* = 13–20).

The lack of an effect of Navβ4 peptide on use-dependent reduction was surprising. In order to further explore this, we also used trains of action potential waveforms to elicit use-dependent reduction. Fig 8A shows the command waveform for the train of 20 slow APs at 33 Hz and representative traces generated in response to the command waveform by hNav1.1 and hNav1.6. Voltage command waveforms for 33 Hz fast AP and 8 Hz slow and fast AP trains can be seen in S1 Fig. The higher frequency trains elicited more use-dependent reduction than low frequency trains (one-way ANOVA; p < 0.05). However, addition of Navβ4 peptide did not significantly impact use-dependent reduction with either hNav1.1 or hNav1.6 at any combination of frequency and AP waveform type (Fig 8B).

## Discussion

In this study we show that hNav1.1 and hNav1.6 exhibit distinct biophysical properties. hNav1.6 has a more hyperpolarized voltage-dependence of steady-state inactivation, faster development of closed-state inactivation, slower kinetics of open-channel inactivation and a greater propensity to generate resurgent currents. The presence of Navβ4 peptide mediated resurgent current generation by hNav1.1 and hNav1.6 allowed for a greater influx of sodium in response to a fast and slow AP waveform with an overall greater sodium influx mediated by hNav1.6. However, Navβ4 peptide did not protect against use-dependent reduction of either channel isoform.

The localization of Nav1.1, Nav1.6 and Navβ4 in the axon initial segment of parvalbumin-positive GABAergic neurons suggests their importance in sustaining high frequency firing. Three critical properties of VGSCs in cells with a fast-firing phenotype that likely contribute to fast-firing include: slow development of inactivation during depolarizing potentials in the inter-spike interval, rapid recovery of channels after an AP spike and incomplete inactivation during APs [40, 41]. Our data indicate that Nav1.1 and Nav1.6 exhibit interesting differences in these potentially crucial biophysical properties suggesting that both channel isoforms are likely to be important for a fast-firing phenotype. The slower development of closed-state inactivation that we observed with Nav1.1 should allow it to be more resistant to inactivation during slow subthreshold depolarizations in the interspike interval compared to Nav1.6. Consequently, more Nav1.1 channels would be available to open once the membrane voltage reaches threshold for firing an AP. In contrast, we would predict that Nav1.6 contributes more to the rapid recovery of channels after an AP spike and incomplete inactivation during APs due to its greater propensity to generate resurgent current since Navβ4 mediated block allows channels to bypass classical inactivation and enhances recovery of the channels. Moreover, Nav1.6 showed faster repriming kinetics at -70mV although Nav1.1 had an overall greater fraction of recovery at voltages ranging from -90mV to -70mV.

Firing frequency correlates strongly with AP width [36, 37]. The width of the AP in many neurons is primarily controlled by repolarization mediated by potassium channels [41, 42]. Our data shows that the width of the AP waveform can greatly alter sodium current kinetics generated by Nav1.1 and Nav1.6. Both channel isoforms produced a larger second non-inactivating component in response to a fast AP waveform, which is consistent with observations in neurons [25, 41]. However, Nav1.6 showed a much greater second non-inactivating component compared to Nav1.1 in response to the fast AP possibly due to its slower rate of inactivation. The duration of the second non-inactivating component in response to the fast AP is increased by Navβ4 peptide for both isoforms although Nav1.6 carried the greatest overall sodium influx. Sodium currents in response to the slow AP waveform produced a smaller second non-inactivating component with both channel isoforms, but Navβ4 peptide increased both the duration as well as the amplitude of the second non-inactivating component. Since the second non-inactivating component occurs during the down stroke of the AP waveform, we would predict that Navβ4 peptide would increase the number of non-inactivated (open) channels available immediately following an AP spike, which may enable burst firing [20]. Notably, the Navβ4 peptide produced a longer duration of increased sodium influx in response to the slow AP compared to the fast AP. This may reflect the ability of Navβ4 peptide to play a more prominent role in neurons with slow AP waveforms. Indeed, in neurons with fast AP waveforms, where the Kv3 family of channels predominantly mediate repolarization, VGSCs channels are primarily protected from inactivation by the fast kinetics of Kv3 channels that force VGSCs to transition directly from open into closed states rather than by open-channel block producing resurgent current [41]. Dynamic clamp studies have shown that Kv3 currents and resurgent sodium currents can synergize to protect channels from inactivation during the interspike interval of spontaneously firing Purkinje neurons [43]. Our data shows that Navβ4 peptide mediates a decrease in sodium current during the initial repolarization phase of the fast waveform (see Fig 5C and 5E). A decrease in sodium flux at this point during a fast AP would likely also synergize with Kv3 currents and contribute to shorter AP durations.

Auxiliary β subunits of VGSCs are known to modulate the biophysical properties of principal α subunits [34, 44]. Navβ4 mediates the generation of resurgent sodium currents and allows channels to bypass classic inactivation. Though Nav1.6 has a greater intrinsic ability to generate resurgent currents, Scn8a knockout animals demonstrate that the contribution of Nav1.6 to resurgent current generation can vary between neuronal populations suggesting that other factors can influence which isoform is predominantly generating resurgent current [16, 45–47]. It has previously been shown that there is a good correlation between resurgent current amplitude and the rate of inactivation, suggesting that Navβ4 competes with the intrinsic inactivation particle to bind the channel pore [33, 48]. Resurgent currents are expected to correlate with enhanced recovery of VGSCs from inactivation [20]. We found that Navβ4 peptide can correspondingly enhance recovery from an open-blocked versus inactivated state. We therefore predicted that Navβ4 peptide would protect channels from undergoing use-dependent reduction, which is the result of channels accumulating into an inactivated state and that this would be greater for Nav1.6 compared to Nav1.1. Unexpectedly, we found that Navβ4 peptide does not protect either Nav1.1 or Nav1.6 from use-dependent reduction with a 10 Hz stimulus. Furthermore, stimulation with trains of fast or slow action potentials also did not uncover a detectable difference in use-dependent reduction in the presence of Navβ4 peptide. Therefore, at least under our experimental conditions, Navβ4 peptide does not appear to compete with the intrinsic mechanisms for use-dependent current reduction. This might be explained in part by proximity of the inactivation particle compared to the diffuse Navβ4 peptide to the channel and thus may not hold true for the full-length Navβ4 (which unfortunately does not produce resurgent sodium currents in HEK293T cells). We therefore tested the full-length Navβ4 in a dorsal root ganglion expression system and did not observe any differences in use-dependent reduction of Nav1.6 currents, suggesting that our results are not a mere artifact of the peptide (data not shown). Indeed, in a recent study comparing striatal neurons from Navβ4 null and wild-type mice, no difference was observed in the number of APs evoked by high-frequency stimulation trains for the two genotypes [49]. However, recent evidence indicates that A-type isoforms of fibroblast growth factor homologous factors (FHFs) mediate fast-onset long-term inactivation of sodium channels in hippocampal neurons [50]. FHFs and Navβ4 have been proposed to differentially regulate neuronal activity [51], and thus we cannot rule out the possibility that Navβ4 might reduce use-dependent current reduction in some neurons by competing with the effect of FHFs on sodium channel inactivation.

Our data shows that hNav1.6 undergoes more use-dependent reduction compared to hNav1.1 and this is consistent with the proposal that Nav1.1 likely plays a major role in maintenance of fast-firing, especially in inhibitory neurons. hNav1.6 currents exhibit slower rates of open-channel inactivation (Fig 4E), larger resurgent currents (Fig 4C) and quicker recovery from open-channel inactivation (Fig 3B) than hNav1.1 currents, indicating that open-channel inactivation is not a major determinant of the use-dependent reduction that we observed. Although slow inactivation might contribute to use-dependent current reduction under some conditions [52, 53], our data and that of others indicate that Nav1.6 channels are less susceptible to slow inactivation than Nav1.1 channels [39]. hNav1.6 channels do exhibit enhanced closed-state inactivation compared to hNav1.1 channels (reflected by a more negative voltage-dependence of inactivation and more rapid development of closed-state inactivation for hNav1.6), suggesting that closed-state inactivation may play an important role in the use-dependent current reduction that we measured.

## Conclusions

In comparison to hNav1.1 channels, hNav1.6 channels display enhanced closed-state inactivation but faster recovery from inactivation, slower open-state inactivation and larger resurgent currents. Interestingly, we did not observe changes in use-dependent current reduction despite the quite large resurgent currents generated in the presence of Navβ4 peptide. Regardless, our study highlights important differences in hNav1.1 and hNav1.6 sodium channel gating properties that are likely to be important in the fine-tuning of neuronal excitability.

## Supporting Information

S1 Fig Waveform command protocol for trains of 33Hz and 8Hz of fast and slow action potentials. (TIF)

S2 Fig Time courses of recovery from fast inactivation at voltages ranging from -70mV to -100mV. (TIF)

S3 Fig Kinetics of fast inactivation in the presence and absence of the Navβ4 peptide for (A) hNav1.1 and (B) hNav1.6. (TIF)

S4 Fig Sodium influx in response to a (A) fast and (B) slow action potential waveform in the absence of the Navβ4 peptide. (TIF)

S5 Fig Recovery from open-blocked (+30mV) and inactivated (-30mV) states in the absence of Navβ4 peptide for (A) hNav1.1 and (B) hNav1.6. (TIF)
