# Supplementary figures and images for: Human Nav1.6 Channels Generate Larger Resurgent Currents than Human Nav1.1 Channels, but the Navβ4 Peptide Does Not Protect Either Isoform from Use-Dependent Reduction
Source: PLoS One. 2015 Jul 16;10(7):e0133485. doi: 10.1371/journal.pone.0133485 (PMC4504674; doi:10.1371/journal.pone.0133485)

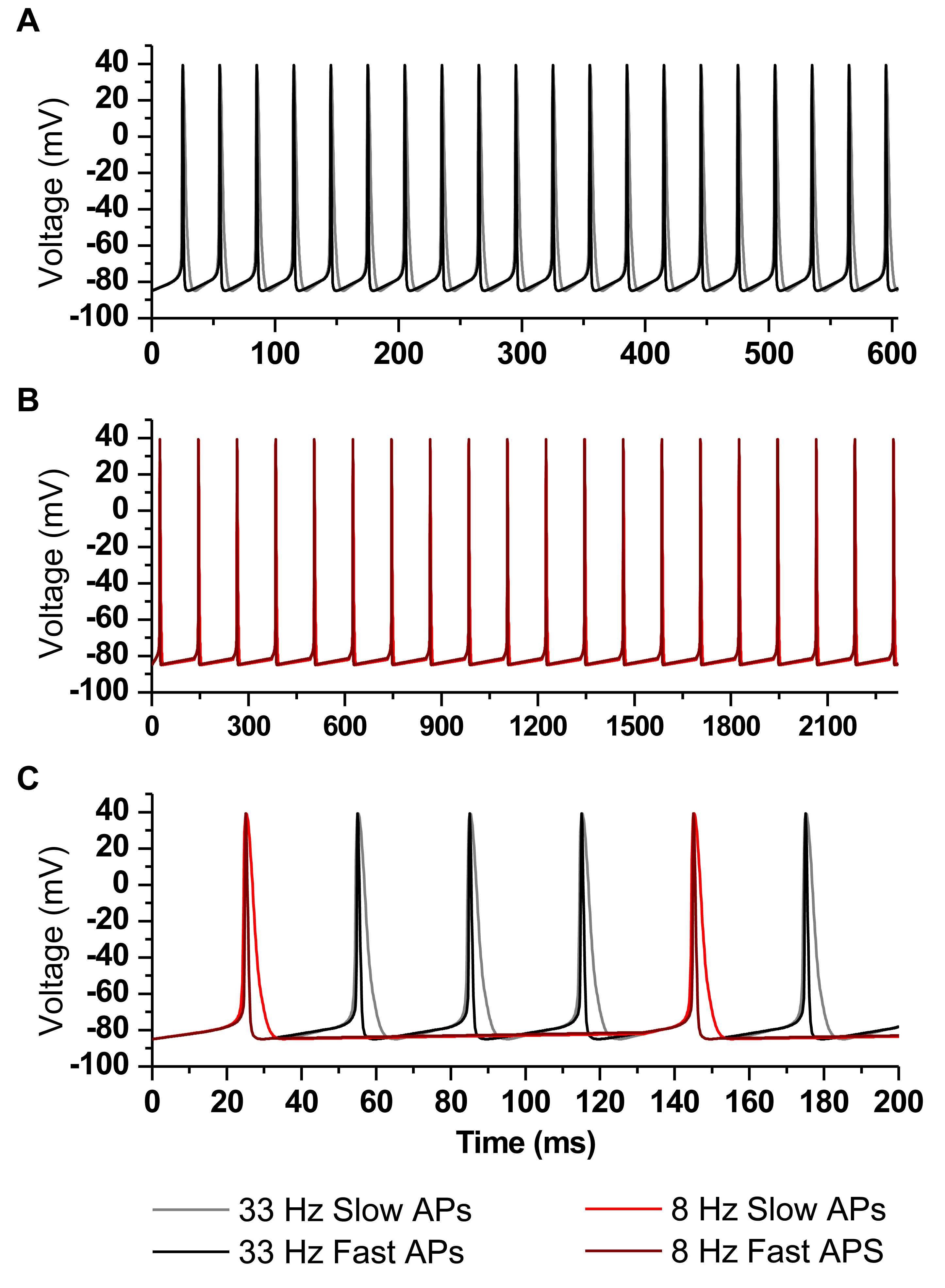

Supplement: S1 Fig — (TIF) [file pone.0133485.s001.tif]

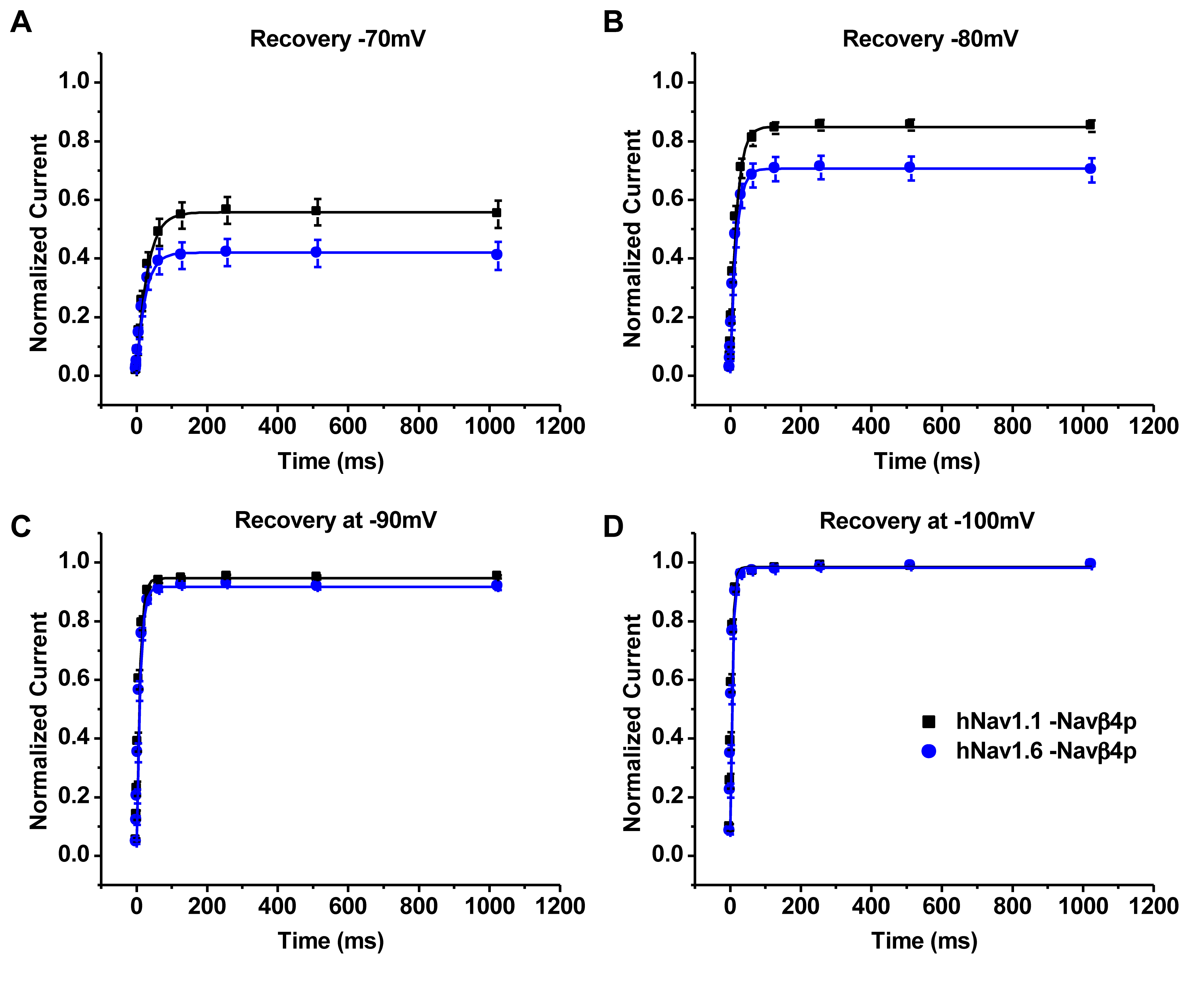

Supplement: S2 Fig — (TIF) [file pone.0133485.s002.tif]

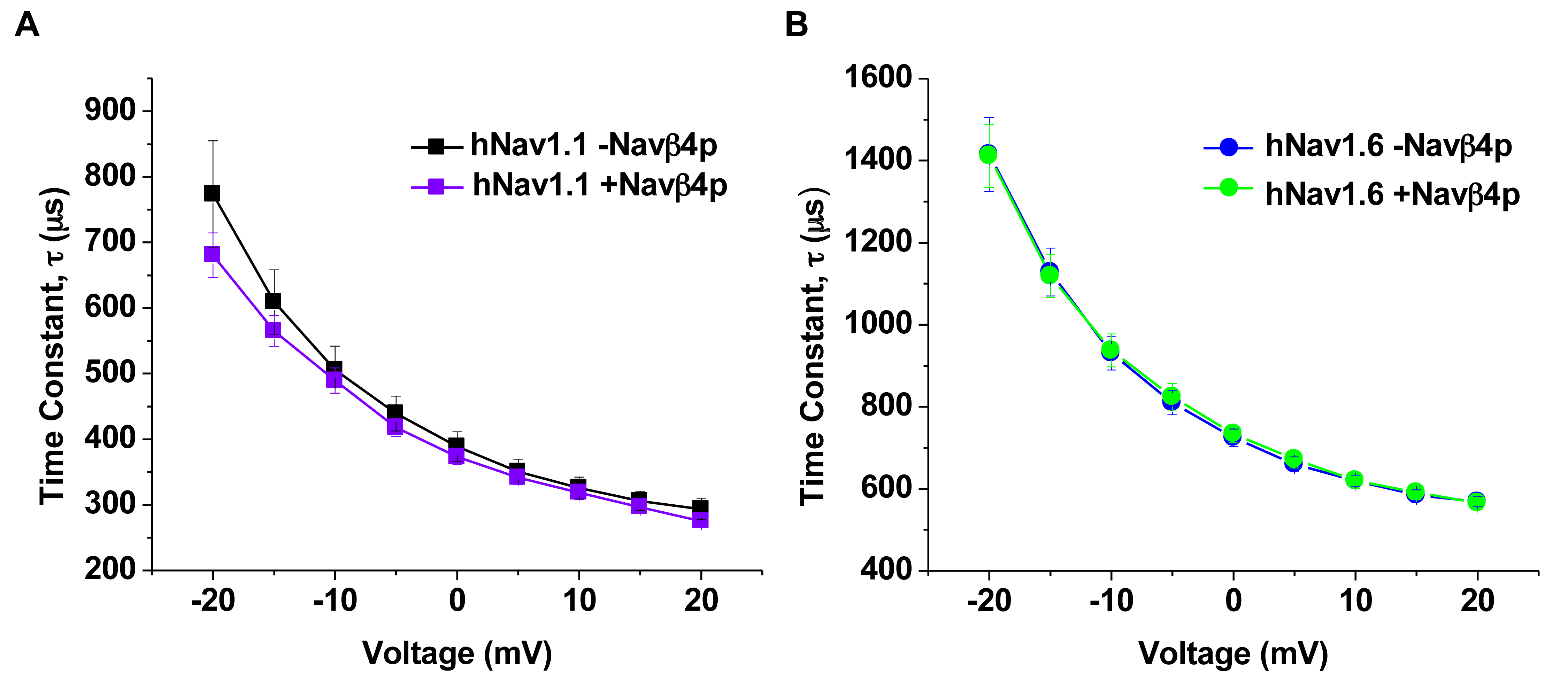

Supplement: S3 Fig — (TIF) [file pone.0133485.s003.tif]

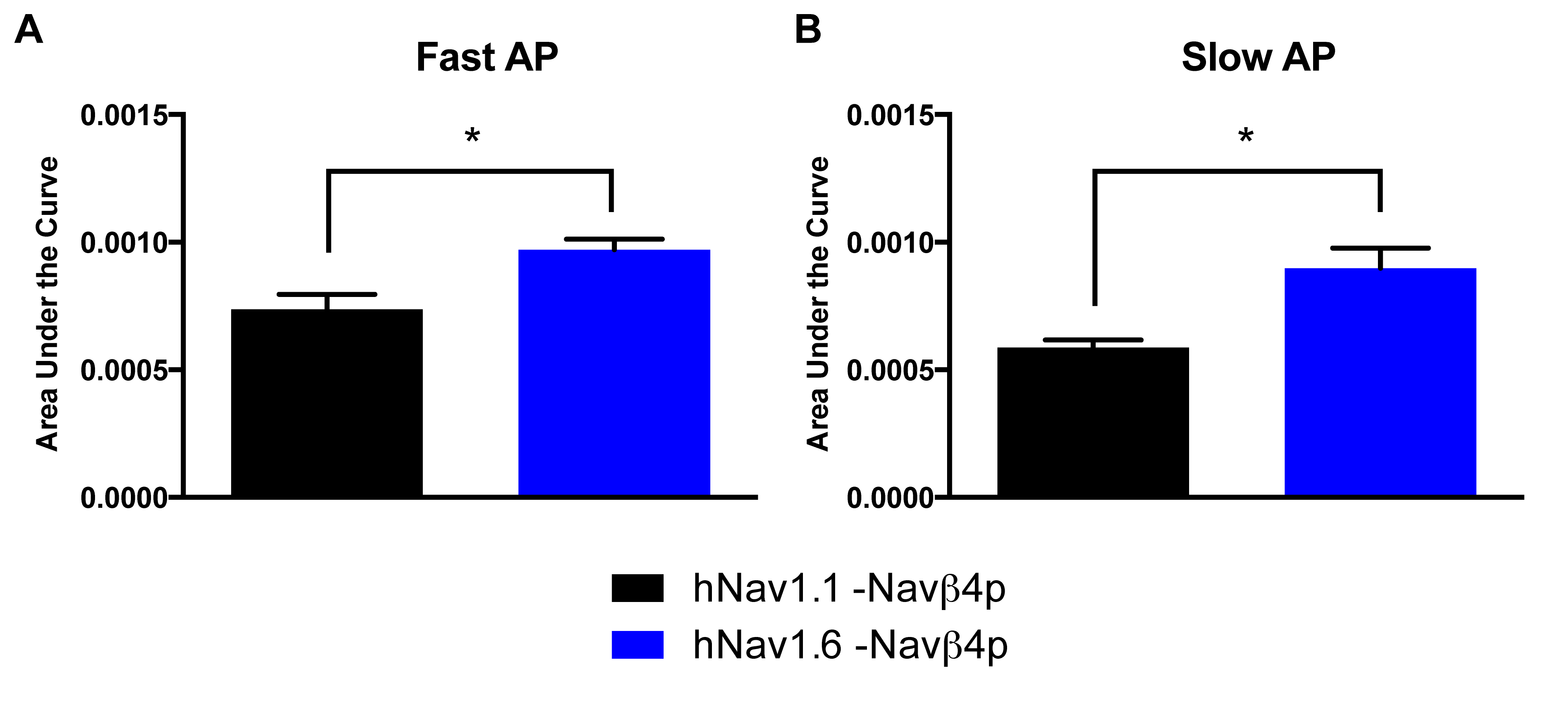

Supplement: S4 Fig — (TIF) [file pone.0133485.s004.tif]

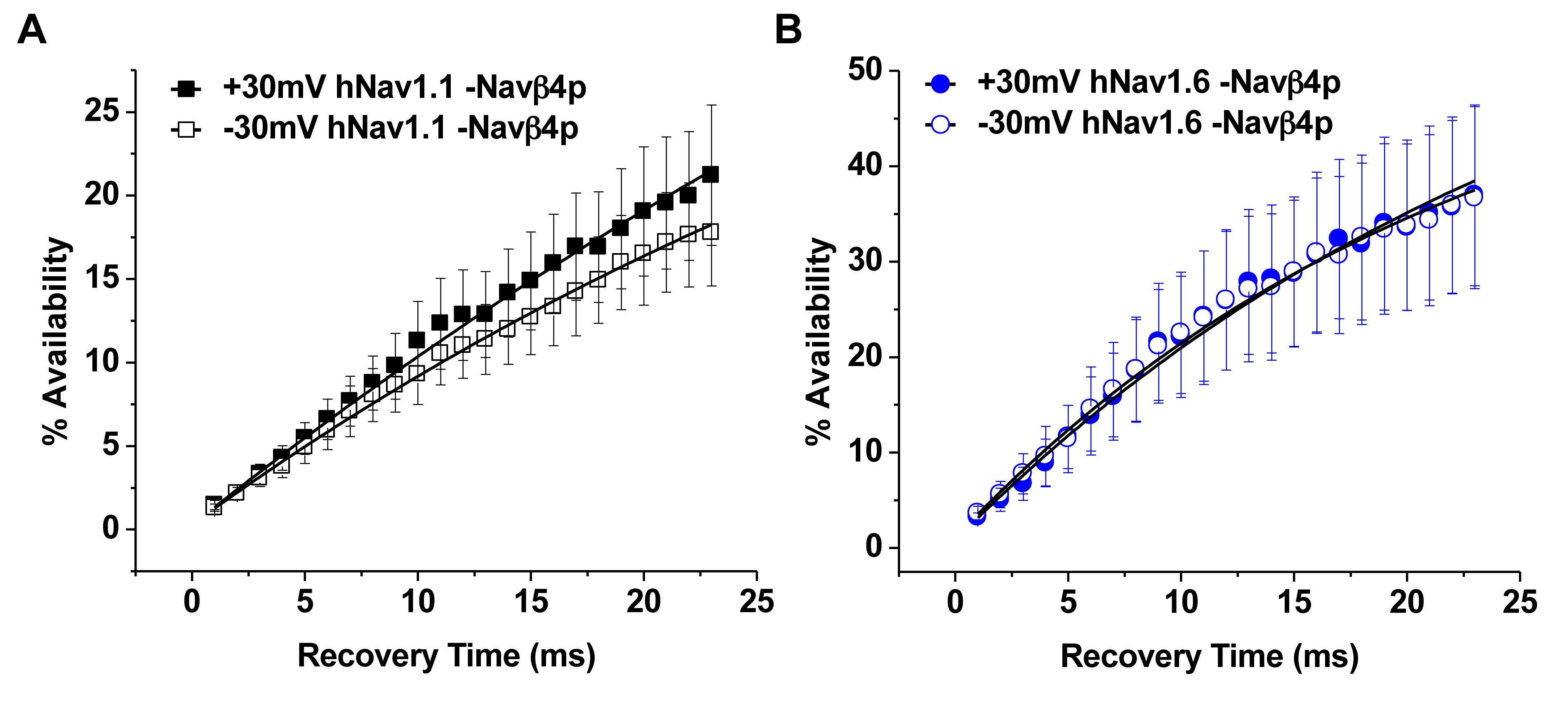

Supplement: S5 Fig — (TIF) [file pone.0133485.s005.tif]
